# Influence of decades-long irrigation with secondary treated wastewater on soil microbial diversity, resistome dynamics, and antibiotrophy development

**DOI:** 10.1016/j.heliyon.2024.e39666

**Published:** 2024-10-22

**Authors:** Amira Yagoubi, Stefanos Giannakis, Anissa Chamekh, Oussama Kharbech, Rakia Chouari

**Affiliations:** a University of Carthage, Laboratory of Plant Toxicology and Environmental Microbiology (LR18ES38), Faculty of Sciences of Bizerte, 7021, Bizerte, Tunisia; b Universidad Politécnica de Madrid (UPM), E.T.S. de Ingenieros de Caminos, Canales y Puertos, Departamento de Ingeniería Civil: Hidráulica, Energía y Medio Ambiente, Environment, Coast and Ocean Research Laboratory (ECOREL-UPM), c/ Profesor Aranguren, 3, ES-28040, Madrid, Spain

**Keywords:** Soil resistome, Wastewater irrigation, Heavy metals, Antibiotics, Antibiotrophy, Resistance gene co-selection

## Abstract

In arid and semi-arid regions, the use of treated wastewater (TWW) for irrigation is gaining ground to alleviate pressure on natural water sources. Despite said treatment, the existing methods fail to eliminate potentially dangerous contaminants. As such, this study assessed the impact of long-term TWW irrigation (5 and 25 years) on soil physicochemical properties and bacterial resistance to heavy metals (Pb, Cu, Cd) and antibiotics (tetracycline and amoxicillin). The results revealed heightened salinity and conductivity and reduced pH in irrigated soils. TWW induces harmful effects by reducing microbial density and size, leading to the disappearance of sensitive populations. Conversely, resilient populations, which mainly utilize antibiotics as a carbon source, have adapted. Metagenomic 16S amplicon sequencing analysis demonstrated a shift, notably reducing *Actinobacteria*, *Bacteroidetes*, and *Firmicutes* while increasing *Acidobacteriota* and *Patescibacteria* in treated soils. Operational Taxonomic Units affiliated with either *Halomonadacea,* or *Saccharimonadacea* and *Vicinamibacteracea*, were defined as indicators of the absence or presence of TWW contamination, respectively. We conclude that TWW irrigation significantly increases bacterial resistance to heavy metals, whereas the impact of antibiotics is nuanced, with antibiotrophy leveraging lower concentrations in treated soils.

## Highlights

• Long-term TWW irrigation significantly altered soil properties and bacterial communities.
• TWW increased soil salinity and conductivity but reduced its pH and microbial density.
• Bacteria developed HM and AR and adapted to TWW exposure using antibiotics as a carbon source.
• Metagenomic analyses showed shifts in the bacterial phyla due to TWW irrigation.
• Indicators, such as Pollution-Induced Community Tolerance, can reveal HM and AR in soils.

## 1 Introduction

The world is facing increasing water scarcity, especially in arid and semiarid regions. Hence, irrigation projects using non-conventional water, particularly treated wastewater (TWW) from sewage treatment plants, are being developed worldwide [1]. It is estimated that 1.32 × 10^6^ m³ of TWW can be reused each year worldwide, 32% of which is intended for agricultural irrigation [2]. In Tunisia, only 2.87 × 10^8^ m^3^ of TWW is reused, and approximately 24% of TWW is used directly for the irrigation of forage crops.

From the perspective of water reuse and equilibrium, TWW reuse in agriculture in Tunisia and other water-deprived regions can be beneficial for the environment, as it reduces its discharge into ecosystems and is rich in nutrients that help plant growth and soil fertility [3,4]. However, TWW is also considered a ‘hotspot’ for the dissemination of bacteria, antibiotics, and heavy metal resistance genes as well as pathogens and complex purifying microbiota, thus comprising a preponderant fraction whose effects are not always known [[5], [6]]. Moreover, the prevalence of multidrug-resistant bacteria is increasing because of environmental pollution and anthropogenic activities. Thus, the terrestrial reservoirs of antibiotic resistance have become one of the most threatening global problems.

It is well known that heavy metals (HM) exert selective pressure on the antibiotic resistance (AR) of terrestrial microorganisms [7] and contaminant resistance occurs through the dissemination of mobile genetic elements through co-resistance, cross-resistance, and co-regulation processes [8]. As antibiotics and heavy metals are present in TWW, it is essential to understand the impacts of these alternative waters on the soil bacterial resistome during short and long periods of soil irrigation. Several studies have shown the cumulative inputs of toxic, highly persistent, and lipophilic pollutants on soil salinization, alkalinity, permeability reduction, and the accumulation of trace metallic elements and nutrients in soil layers [9]. Furthermore, with soil leaching and entrainment of potentially toxic elements and nutrients deep into soils, consequences on groundwater are not negligible and can directly affect soil microbial diversity and activity, as well as global health [10,11].

While some authors have suggested that TWW affects AR levels in the soil microbiome [12,13], surprisingly, other studies have shown identical or higher levels of antibiotic resistance genes (ARG) in freshwater-irrigated soils than in TWW-irrigated soils. These results suggest that antibiotic-resistant bacteria (ARB) that entered the soil from the TWW did not survive, are probably not competitive, and that these resistant bacteria are predominantly associated with the native soil resistome [14,15]. Clues about this contradiction could be brought by further studies on water and soil quality analyses.

To address this issue and contribute to the understanding of the existing dichotomy in the literature, this study aimed to analyze the effects of TWW irrigation in agriculture. We specifically examined the long-term effects of TWW irrigation on the soil properties, bacterial communities, and their resistomes. The focus was on HM resistance, using copper (Cu), lead (Pb), and cadmium (Cd) as representative toxic metals, as well as tetracycline (TET) and amoxicillin (AMX) as model antibiotics. Additionally, we investigated how the co-occurrence of these stressors influenced the development of resistance. We will investigate whether treated wastewater (TWW) can lead to increased antibiotic and/or heavy metal resistance in soil bacteria using traditional culturing techniques, selective pressure experiments, and metagenomic analysis. This research will help guide best practices for wastewater reuse.

## 2 Materials and methods

### 2.1 Soil sampling sites

Soil was sampled from the Cebela Borj Touil perimeter**,** the largest area irrigated by TWW in Tunisia, covering approximately 3145 ha. Soil sampling was carried out in March 2020, and three plots occupied by fodder crops were considered.

1) Site S1 (36°56′35.1″ N, 10°08′10.8″ E): Plot S1 was never irrigated with TWW and serves as a control.
2) Site S2 (36°56′52.5″ N, 10°7′23.9″ E): Plot S2 is irrigated with TWW for 5 years; and
3) Site S3 (36°56′52′20.7″” N, 10°08′38.0” ″ E): Plot S3 has been irrigated with TWW for the last 25 years.

TWW originates from three wastewater treatment stations (Charguia, Choutrana I, and the North Coast) based on the activated sludge process, and is discharged to the sea by a transfer chain formed by Canal El Khalij and Oued El Khalij. One pumping station takes TWW from the Canal El Khalij and discharges it into a regulation basin. The latter provides farmers with TWW necessary for irrigation [16]. HM concentrations in sampled TWW were 0.992 ± 0.07 mg/L, 1.248 ± 0.1 mg/L for Cu and Pb respectively while Cd was not detected. TWW physico-chemical parameters are shown in Supplementary Table S1.

Soil samples were collected from the surface layer (at a depth of 40 cm) using an auger. The soil cores were collected in sterile bags and placed in a cooler for transport. Finally, in the laboratory, the samples were aliquoted and kept at −20 °C for molecular biology experiments, at 4 °C for bacterial enumeration, or dried at room temperature after sieving (2 mm) for physicochemical measurements.

### 2.2 Physicochemical characteristics

For each sample, 10 g of soil was mixed with 50 mL sterile distilled water. After stirring overnight (70 g/min) using a Platform Shaker STR6, the samples were centrifuged at 1082×*g* for 10 min. The supernatants were recovered and filtered [17]. The leachates were subjected to physicochemical analysis (pH and electrical conductivity (EC, in dS/m) measurements) using a Multi 3320 SET 1. Thus, salinity was calculated from electrical conductivity [18], according to Eq. (1):

Eq. 1 Salinity=EC∗640

With a correction factor of 640, according to the international convention, 1 dS/m is equivalent to 640 mg/L mixed salts.

### 2.3 Heavy metals measurements

For these tests, 1 g of soil was subjected to acid digestion with 1:1:1 HNO_3_, 30 % H_2_O_2_, and concentrated HCl at 95 °C (reagents were purchased from Sigma-Aldrich). After cooling, the digested samples were filtered and brought to a final volume of 10 mL with distilled water [19].

The samples were analyzed by inductively coupled plasma optical emission spectrometry (ICP-OES). The metals analyzed were Al, As, Cu, Pb, Mn, Cd, Se, Fe, and Zn.

### 2.4 Enumeration of total bacterial aerobic mesophilic flora

For each sample, 0.4 g of soil was washed twice with 0.8 mL of 1X PBS (Phosphate Buffer Saline 0.01 M), vortexed (1 min), and centrifuged for 5 min at 8832×*g*. Sterile distilled water (10 mL) was added to the pellet obtained from each soil sample, and the homogeneous suspension obtained constituted the 10^−1^ dilution. Serial ten-fold dilutions were obtained from 10^−2^ to 10^−5^. Next, 100 μL of each dilution was spread on Petri dishes containing Plate Count Agar (PCA) medium. For each dilution, three plates were spread (surface seeding) with bacterial suspension [20].

Colonies were counted after 24 h of incubation at 30 °C, and the results were expressed as the number of colony-forming units per gram (CFU/g) [21] of soil according to the following formula (Eq. (2)):

Eq. 2 $$\left[N\right]=\frac{\sum C\;}{\left(v*1.1d\right)}$$

With:

Σ C: average of all colonies counted on 2 successive plated aliquots.

V: inoculum volume applied on each Petri dish (in mL)

d: dilution rate of the highest dilution used for dish counts.

### 2.5 Enumeration of total bacterial aerobic mesophilic flora resistant to heavy metals

To test the effect of TWW on the HM resistance of microorganism samples, stock solutions were prepared using copper (CuSO_4_), lead (Pb (NO_3_)_2_), and cadmium (CdCl_2_). The metal salt concentration was defined after Minimum Inhibitory Concentration (MIC) determination.

The prepared stock solutions were then sterilized using a 0.45 μm diameter filter (Millipore). Finally, they were kept in 50 ml sterile vials at 4 °C in the dark until use.

Two concentrations of HM near the MIC) (0.5 g/L and 1 g/L for copper and lead: 0.1 g/L and 0.3 g/L for cadmium) are tested to study their effects on the soil bacterial community. The counting method described above (Section 2.4) was used after adding an appropriate volume of the desired metal solution. Bacterial colonies were counted after 24 h of incubation at 30 °C, and the results were expressed as CFU/g of soil.

### 2.6 Enumeration of total bacterial aerobic mesophilic flora resistant to antibiotics

The concentrations used to assess the effect of TWW on antibiotic resistance in microorganisms were selected according to Glibota et al. [22]. The plate count method detailed in section 2.4 was applied here on PCA plates with TET (2 mg/L; Sigma-Aldrich) and AMX (64 mg/L; Sigma-Aldrich). The plates were incubated for 24 h at 30 °C, after which colony-forming units (CFUs) were counted and reported as CFU per gram of soil. PCA plates without antibiotics served as controls for each soil sample.

### 2.7 Investigation of co-occurrence between heavy metals and antibiotic resistance in bacterial isolates

The tested bacterial strains (x18) were isolated from the soil samples (S1, S2, and S3) on Brain Heart Infusion (BHI) plates (Table 3). After purification, they were stored in glycerol stock at -20 °C and revived on BHI agar plates after overnight incubation at 30 °C.

Four combinations of HMs and ABs were tested on bacterial strains to investigate the potential co-occurrence of trace metals and antibiotic resistance. The combinations were (Cu + TET), (Cu + AMX), (Pb + TET), and (Pb + AMX).

For the experiment, the following control and test conditions were used.

• **Positive control:** one bacterial strain grown in liquid BHI medium without heavy metals or antibiotics.
• **Negative control:** liquid BHI medium containing HM + AB without bacterial inoculation.
• **Test conditions:** liquid BHI medium containing HM + AB inoculated with one bacterial strain.

The concentration of the heavy metals (Cu and Pb) was set at 0.5 g/L, a level determined to allow optimal bacterial growth. The antibiotic concentrations used were identical to those in section 2.6 (2 mg/L and 64 mg/L for TET and AMX, respectively). Each bacterial strain was exposed to HM + AB and the cultures were incubated at 30 °C for 24 h. The growth response was observed to assess any co-occurrence effects between trace metals and antibiotic resistance in the bacterial isolates.

### 2.8 Antibiotrophy test

This test allowed us to determine whether the AR strains present in the three studied soils could use AB as a carbon source. The strains were obtained from all 3 sampling sites. The medium used to grow the bacteria has been described previously by Dantas et al. [23] and Yagoubi et al. [24]. The pH was adjusted to 5.5, and the medium was sterilized using a 0.22 μm filter. The Erlenmeyer flasks were incubated at 30 °C. The OD at 600 nm was measured every 8 h using a spectrophotometer (Shimadzu UV-1800, Japan) for two days. These measurements are then presented using a growth curve plotting OD_600_ = f(t). Three controls were used (MM + soils; MM + Tet and MM + AMX)

### 2.9 16S rRNA gene soil metagenome sequencing and analyses

The composition and structure of the sampled microbial communities were assessed through amplification and sequencing of the V3-V4 variable regions of the 16S rRNA gene using the following forward and reverse primers: 5′-CCTACGGGNGGCWGCAG-3′ 5′-GACTACHVGGGTATCTAATCC-3’ [25]. The reaction was carried out in 50 ml volumes containing 0.3 mg/ml BSA (Bovine Serum Albumin), 250 μM dNTPs, 0.5 μM of each primer, 0.02 U of Phusion High-Fidelity DNA Polymerase (Finnzymes OY, Espoo, Finland), and 5x Phusion HF Buffer containing 1.5 mM MgCl₂. The following PCR conditions were used: initial denaturation at 95 °C for 5 min, followed by 25 cycles of denaturation (95 °C for 40 s), annealing (55 °C for 2 min), and extension (72 °C for 1 min). The final extension step was performed at 72 °C for 7 min. The PCR products were purified using the QiaQuick PCR Purification Kit (QIAGEN, Hilden, Germany). Negative controls for PCR and positive Mock Community controls were included to ensure quality control. Raw demultiplexed forward and reverse reads were processed using QIIME2 [26]. Illumina MiSeq 300 × 2 sequencing was performed using Microomics software (Microomics Systems S.L., Spain). Sequence analysis involved several key steps: primer trimming, quality filtering, denoising, pair-end merging, and phylotype calling, all of which were performed using the DADA2 method [27]. Phylogenetic assessment was conducted using Mafft [28] and Fast Tree [29]. Alpha diversity metrics, including observed OTUs and Pielou's evenness, were calculated from the phylotype data. Additionally, beta diversity was derived from both the phylotype and phylogenetic data. Taxonomic assignment was achieved using a Bayesian Classifier trained with the Silva database version 138 (99 % OTUs full-length sequences) [30]. This comprehensive bioinformatics approach enabled a detailed analysis of the microbial community structure and diversity.

### 2.10 Statistical analysis

Variations in enumeration, physicochemical characteristics, and the concentration of trace metallic elements among the different soil samples were analyzed using analysis of variance (ANOVA) with the Tukey HSD post-hoc test, performed with STATISTICA software (p < 0.005).

## 3 Results

### 3.1 Impact of TWW irrigation on soil characteristics: pH, salinity, and electrical conductivity

Soil S1, which had never been irrigated with treated wastewater (TWW), had a pH of 7.93 (±0.11). In contrast, soil samples irrigated with TWW exhibited slightly lower pH values: 7.61 (±0.35) for soil irrigated for 5 years (S2) and 7.17 (±0.54) for soil irrigated for 25 years (S3). Electrical conductivity was higher in soils irrigated with TWW compared to the control, with values of 1.34 dS/m for S1, 2.92 dS/m for S2, and 3.85 dS/m for S3. Consequently, salinity levels were elevated in TWW-irrigated soils, reaching up to 1.57 mg/L, 1.87 mg/L, and 2.46 mg/L for S1, S2, and S3, respectively (Table 1). Additionally, there was an accumulation of all heavy metals (HMs) studied in the soils with increasing duration of irrigation (Table 2). For example, the zinc concentrations were 10.36 mg/kg in S1, 53.25 mg/kg in S2, and 60.75 mg/kg in S3.

**Table 1:** Variation in physicochemical parameters (pH, electrical conductivity, and salinity) of leachate from TWW-irrigated soils within the Cebela-Borj Touil perimeter.

		S1				S2				S3			
	Unit	Min	Max	M	SD	Min	Max	M	SD	Min	Max	M	SD
**pH**	Standard	7.8	8.02	**7.93**	0.11	7.21	7.83	**7.61**	0.35	6.61	7.68	**7.17**	0.54
**EC**	dS/m	1.26	1.41	**1.34**	0.07	2.84	3.01	**2.92**	0.09	3.71	4.11	**3.85**	0.22
**Salinity**	mg/L	1.54	1.60	**1.57**	0.03	1.81	1.92	**1.87**	0.05	1.99	3.01	**2.46**	0.51

Min, minimum; Max, maximum; M, mean; S, standard deviation; EC, electrical conductivity. S1: soil not irrigated with TWW; S2: soil irrigated for 5 years with TWW; S3: soil irrigated for 25 years with TWW. (The results are presented as mean ± standard error of three independent repetitions.).

**Table 2:** Se, Zn, Mn, Cd, As, Pb, and Al concentrations in soil samples (S1, S2, and S3).

Soil	Se	Zn	Mn	Cd	As	Pb	Al
				mg Kg^−1^			
**S1**	0.94^a^±0.02	10.35^a^ ±2.00	0.075^a^ ±0.02	0.005^a^ ±0.001	0.034^a^ ±0,02	0.945^a^ ±0.2	11.04^a^ ±1.4
**S2**	1.06^a^ ±0.14	53.25^b^ ± 1.41	2.39^b^ ± 0.14	0.025^b^ ± 0.007	0.395^b^ ± 0,07	1.098^ab^ ± 0.14	16.23^b^ ± 1.41
**S3**	1.34^b^ ± 0.28	60.75^c^ ±1.41	4.89^c^ ±0.29	0.055^b^ ± 0.021	0.505^b^ ± 0.16	1.245^b^ ± 0.01	16.28^b^ ± 1.77

a-c: For each parameter, different letters indicate significant differences at p < 0.05, according to Tukey's test.

The results are presented as mean ± standard error of three independent repetitions.

**Table 3:** Resistance profiles to metals (Pb, Cu, and Cd), antibiotics (amoxicillin and tetracycline), and co-selection with metallic trace elements and antibiotics of different strains isolated from soils (S1, S2, and S3).

		Heavy Metals			Antibiotics		Co-occurrence			
Strains	*Origin*	*Pb (*1 g/L*)*	*Cu (*1 g/L*)*	*Cd (0.*3 g/L*)*	*AMX (*64 mg/L*)*	*TET (*2 mg/L*)*	*Cu + AMX*	*Cu + TET*	*Pb + AMX*	*Pb + TET*
**1**	***S1***	+++	++-	–	–	++-	–	++-	+++	++-
**2**	***S1***	–	–	+++	++-	+++	++-	+++	–	+++
**3**	***S1***	–	++-	+++	+++	–	+++	++-	+++	–
**4**	***S3***	++-	+++	++-	+++	+++	++-	++-	+++	+++
**5**	***S2***	+++	+++	++-	+++	+++	++-	–	+++	++-
**6**	***S2***	–	++-	–	+++	+++	++-	+++	+++	+++
**7**	***S3***	+++	+++	+++	+++	+++	++-	++-	+++	++-
**8**	***S2***	+++	+++	++-	+++	+++	–	+++	+++	+++
**9**	***S3***	+++	+++	+++	+++	+++	+++	+++	+++	+++
**10**	***S3***	+++	++-	++-	+++	+++	+++	+++	+++	+++
**11**	***S1***	+++	+++	–	–	++-	++-	+++	+++	–
**12**	***S2***	+++	++-	–	++-	+++	–	++-	++-	+++
**13**	***S3***	++-	++-	+++	++-	+++	++-	++-	++-	+++
**14**	***S1***	–	–	+++	++-	++-	++-	–	+++	–
**15**	***S2***	++-	+++	–	+++	+++	+++	+++	+++	+++
**16**	***S2***	++-	+++	–	+++	+++	++-	++-	+++	++-
**17**	***S1***	–	++-	–	–	+++	–	+++	–	–
**18**	***S3***	+++	+++	++-	++-	+++	+++	++-	++-	+++

---: no growth; ++-: 0.6 < DO_600nm_ < 1.2; +++: DO_600nm_ > 1.2.

### 3.2 Enumeration of total mesophilic aerobic flora according to TWW soil irrigation duration

**Fig. 1:**
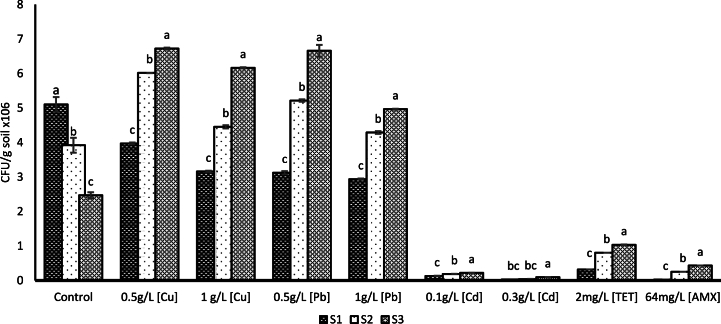
Enumeration of total mesophilic aerobic flora in TWW-irrigated soil samples in the presence of Cu, Pb, Cd, TET, and AMX (S1: control soil never irrigated with TWW; S2: soil irrigated for 5 years; S3: soil irrigated for 25 years with TWW). The results are presented as mean ± standard error of four independent repetitions. a–c: For each parameter, different letters indicate significant differences at p < 0.05, according to Tukey's test.

The typical bacterial count in soil that had not been irrigated with TWW was approximately 5.1 × 10^6^ (±2.1 × 10^5^) CFU/g. In contrast, soils irrigated with TWW for 5 years and 25 years had reduced bacterial counts, with values of 3.9 × 10^6^ (±2.1 × 10^5^) CFU/g and 2.5 × 10^6^ (±8 × 10^4^) CFU/g, respectively (Fig. 1 ‘control’).

### 3.3 Enumeration of total bacterial aerobic mesophilic flora resistant to heavy metals

In the presence of Cu, Pb, and Cd, bacterial counts were significantly reduced compared to those in the control. However, the total number of bacteria resistant to Cu, Pb, and Cd was higher in soils that had been irrigated with TWW for the longest period. In addition, the higher the HM concentration in the soil, the lower the bacterial concentration. (Fig. 1). At a concentration of 0.5 g/L Cu added to the PCA medium, the number of bacteria present in S1, S2, and S3 was estimated at 4 × 10^6^ (±2.8 × 10^4^), 6 × 10^6^ (±0.57 × 10^4^), and 6.7 × 10^6^ (±2.8 × 10^4^) CFU/g soil, respectively. At a concentration of 1 g/L Cu added to the medium, 3.2 × 10^6^(±2.3 × 10^4^), 4.4 × 10^6^(±4.5 × 10^4^), and 6.2 × 10^6^ (±1.7 × 10^4^) CFUs/g of soil were counted. Similarly, at a concentration of 0.1 g/L Cd added to the growing medium, the number of bacteria present in the S1, S2, and S3 soils was estimated to be 1.3 × 10^5^ (±0.57 × 10^3^), 1.9 × 10^5^(±0.57 × 10^3^), and 2.2 × 10^5^ (±0.57 × 10^3^) CFU/g of soil. At a concentration of 0.3 g/L Cd added to the medium, 2.8 × 10^4^(±0.57 × 10^3^), 4 × 10^4^(±0.57 × 10^3^), and 9.6 × 10^4^ (±0.57 × 10^3^) CFU/g of soil were counted.

Indeed, with the addition of 0.5 g/L of Pb to the culture medium, the number of bacteria in soils S1, S2, and S3 was estimated at 3.1 × 10^6^ (±4.5 × 10^4^), 5.2 × 10^6 (±3.4 × 10^4^), and 6.7 × 10^6^ (±1.7 × 10^5^) CFU/g of soil, respectively. When the Pb concentration was increased to 1 g/L, the bacterial counts were 2.9 × 10^6^ (±1.7 × 10^4^), 4.3 × 10^6^ (±4.0 × 10^4^), and 5.0 × 10^6^ (±1.1 × 10^4^) CFU/g of soil for S1, S2, and S3, respectively.

### 3.4 Enumeration of total bacterial aerobic mesophilic flora resistant to antibiotics

The results for AR biomass are shown in Fig. 1. In the presence of tetracycline, the longer the duration of irrigation of the soil with TWW, the higher the number of TET-resistant bacteria compared to the control. Thus, in the presence of 2 mg/L TET added to the culture medium, the number of bacteria present in soils S1, S2, and S3 was estimated to be 3.2 × 10^5^ (±0.8 × 10^3^), 8 × 10^5^ (±0.8 × 10^3^), and 10^7^ (±1.3 × 10^3^) CFU/g soil, respectively. Similarly, a significant increase in the number of AMX-resistant bacteria was observed with the duration of irrigation with TWW. In fact, with the 64 mg/L of AMX added to the culture medium, the number of ARB was estimated at 2.2 × 10^5^ (±1.1x103), 2.5 × 10^5^ (±0.8 × 10^3^), and 4.3 × 10^5^ (±0.8 × 10^3^) CFU/g of soil in S1, S2, and S3, respectively. In addition, TET-resistant bacteria appeared to be more abundant than AMX-resistant bacteria.

### 3.5 Tolerance results

#### 3.5.1 Metallic trace element tolerance determination

To assess the tolerance of the selected strains to various trace metals (Cu, Pb, and Cd), each bacterial strain was grown in a BHI liquid medium containing a specific metal. The maximum MIC values recorded for Pb, Cd, and Cu were 1 g/L, 0.3 g/L, and 1 g/L, respectively.

The prevalence of multi-resistance to all three metals was higher than that of double- or single-resistance (Table 3), with 44.4 % of the strains showing multi-resistance, 33.3 % exhibiting double resistance, and 27.7 % showing single resistance. Single resistance was particularly common in strains isolated from S1 soil, with 66.6 % of the strains being mono-resistant compared to 16.6 % from S2 and 0 % from S3. Moreover, double resistance was expressed in a high proportion of the strains in soil S2 (66.6 %) and S1 (16.6 %) compared to that in soil S3 (0 %). Thus, it is very interesting that the proportion of microorganisms possessing multi-resistance was more frequent in S3 (66.6 %) than in S2 (33.33 %), and 0 % for S1. Moreover, the frequency of Cu-resistant strains (89 % of all strains) was higher than those of Pb (72 %) and Cd (61 %).

#### 3.5.2 Antibiotic tolerance determination

Two antibiotics were used to evaluate the resistance of the bacterial strains isolated from various plots irrigated with treated wastewater. The resistance frequency to AMX was approximately 83 %, while the resistance to TET was notably higher, with 94.5 % of the strains exhibiting resistance. In addition, 77.8 % of the strains were resistant to both AMX and TET.

#### 3.5.3 Analysis of co-occurrence of metallic trace elements and antibiotic resistance

The co-selection of pollutant resistance in the different selected strains was verified by incubating each bacterial isolate in the simultaneous presence of metal salts and ABs. Bacterial strains isolated on Pb-containing media demonstrated 77.7 %, 88.9 %, and 72.3 % resistance to TET, AMX, and AMX + TET, respectively. Conversely, fewer strains isolated from the Cu-containing media were resistant to AMX (77.8 %), TET (88.9 %), and AMX + TET (66.7 %). We observed that multi-tolerance to Cu and Pb was significantly associated with microbial multi-resistance to TET and AMX (44.4 %).

### 3.6 Antibiotrophy test

**Fig. 2:**
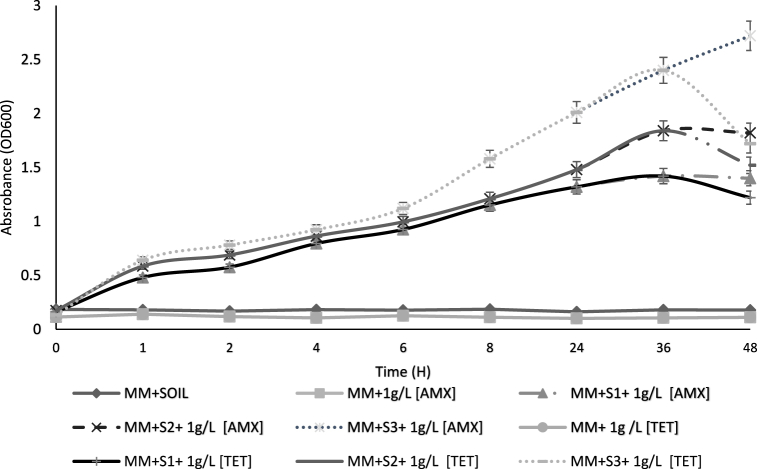
Growth of strains isolated from S1, S2, and S3 in the presence of 1 g/L TET, and 1 g/L AMX in mineral medium for 2 days. The results are presented as mean ± standard error of four independent repetitions.

The growth capacity of the three soil strains in mineral medium supplemented with TET and AMX is illustrated in Fig. 2. The addition of 1 g/L of TET resulted in a significant increase in bacterial growth, with peak optical densities at 600 nm reaching 1.22, 1.52, and 1.7 for strains S1, S2, and S3, respectively, after 48 h of incubation at 30 °C. These results indicated that TET substantially promoted bacterial proliferation in the medium. At the same time, we noticed the ability of the isolates to develop in a mineral medium with 1 g/L of AMX (Fig. 2). After 48 h at 30 °C, the OD_600_ values of S1, S2, and S3 were 1.40, 1.82, and 2.72 respectively. The bacterial microflora from S3 showed the best capacity to grow in the mineral medium in the presence of TET and AMX, followed by those from S2 and finally from S1 (Fig. 2).

### 3.7 Impact of TWW irrigation on soil bacterial communities

Metagenomic sequencing of the 16S rRNA gene was performed to determine the diversity of bacterial phylogenetic groups in the different soil samples irrigated with TWW. After the quality control check, 63337 sequences were analyzed and grouped into 276 Operational Taxonomic Units (OTUs) (and 516 phylotypes corresponding to amplicon sequence variant calls by the DADA2 pipeline). OTUs were independent of relative abundance; however, singletons and doubletons were excluded. The native soil microbiota was composed of 114, 129, and 148 bacterial OTUs among the 23575, 20287, and 19475 sequences for S1, S2, and S3 soils, respectively. Shannon indices of 5.803, 5.932, and 6.047 were obtained for S1, S2, and S3 soils, respectively (Table 4). These results indicate that the samples have an important bacterial diversity, which is practically the same or even slightly increased with irrigation time. These results were confirmed by Pielou evenness, which measures diversity along with species richness. For the three soil samples, the index was close to 1, indicating that the OTUs present in the samples had identical abundance. Phylogenetic analysis grouped sequences into 19 phyla, 46 classes, 91 orders, 133 families, and 181 genera.

**Table 4:** Taxonomic diversity indices calculated for the three studied soils (S1: control; S2: irrigated with TWW for 5 years; S3: irrigated with TWW for 25 years). Richness (S), expressed as the number of OTUs, and alpha diversity indices with Pielou evenness (J′) and Shannon indices (H′) are displayed.

Soil	Number of OTUs (S)	Pielou's evenness (J′)	Shannon index (H′)
**S1**	114	0.90402	5.803
**S2**	129	0.88688	5.932
**S3**	148	0.86927	6.047

**Fig. 3:**
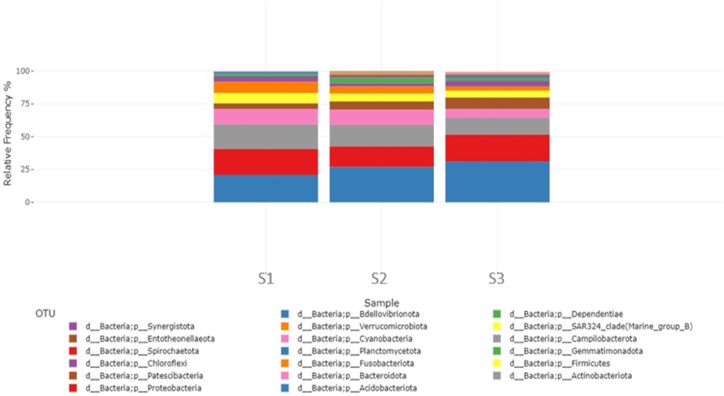
Bar plots of relative abundance at the phylum level found in soils never irrigated with wastewater (S1), soils irrigated for 5 years (S2), and soils irrigated for 25 years (S3), based on relative frequency %.

Fig. 3 shows the relative frequency of the bar plot at the phylum level. In the S1 soil, which was never irrigated with TWW, the *Acidobacteriota* phylum was overrepresented with 20.7 % of the total sequences, followed by *Proteobacteria* (19.8 %), *Actinobacteriota* (18.9 %), *Bacteroidota* (11.5 %), *Fusobacteriota* (8.6 %), *Firmicutes* (7.8 %), *Patescibacteria* (4.4 %), and *Chloroflexi* (4.2 %). *Gemmatimonadota*, *Planctomycetota*, *Campilobacterota*, and *Cyanobacteria* were less represented (<2 % of the total sequences).

In the S2 sample, which was a soil irrigated for 5 years with TWW, *Acidobacteriota* was the most abundant phylum, accounting for 26.9 % of the total sequences, followed by *Actinobacteriota* (16.4 %), *Proteobacteria* (15.6 %), *Bacteroidota* (11.7 %), *Patescibacteria* (6.3 %), *Firmicutes* (5.9 %), *Fusobacteriota* (5.5 %), *Gemmatimonadota* (4.1 %), and *Chloroflexi* (2.5 %). *Campilobacterota*, *Spirochaetota*, *Planctomycetota*, *Verrucomicrobiota*, *Entotheonellaeota*, *Dependentiae*, *SAR324_clade*, *Cyanobacteria*, and *Synergistota* accounted for less than 2 % of the total sequences (Fig. 3).

In the S3 sample, which was a soil irrigated for 25 years with TWW, *Acidobacteriota* was the most abundant phylum, accounting for 31 % of the total sequences, followed by *Proteobacteria (*20.4 %), *Actinobacteriota (*12.9 %), *Patescibacteria* (8.8 %), *Bacteroidota* (6.7 %), *Firmicutes* (5.2 %), *Chloroflexi* (4.3 %), *Fusobacteriota* (3.1 %), *Gemmatimonadota* (2.1 %), and *Planctomycetota* (1.7 %). *Cyanobacteria*, *Spirochaetota*, *Campilobacterotat*, *SAR324_clade (Marine_group_B)*, *Entotheonellaeota*, and *Bdellovibrionota* represented by less than 1 % of the total sequences (Fig. 3).

Within the *Acidobacteriota* phylum, *Vicinamibacterales* was the most represented order, accounting for 19.8 %, 26.2 %, and 30.1 % of the total sequences in S1, S2, and S3, respectively, followed by the *Fusobacteriales* order within the *Fusobacteriota* phylum, accounting for 8.6 %, 4.5 %, and 3.1 % of the total sequences, respectively. Within *Gammaproteobacteria*, the order *Diplorickettsiales* was more represented in S3 (8.2 %) than in S2 (4.8 %) or S1 (1 %), particularly in the genus *Aquicella*. Within the phylum *Actinobacteria*, the *Corynebacteriaceae* family was represented by 5 %, 3,2 %, and 3.2 % of all sequences in S1, S2, and S3, respectively.

**Fig. 4:**
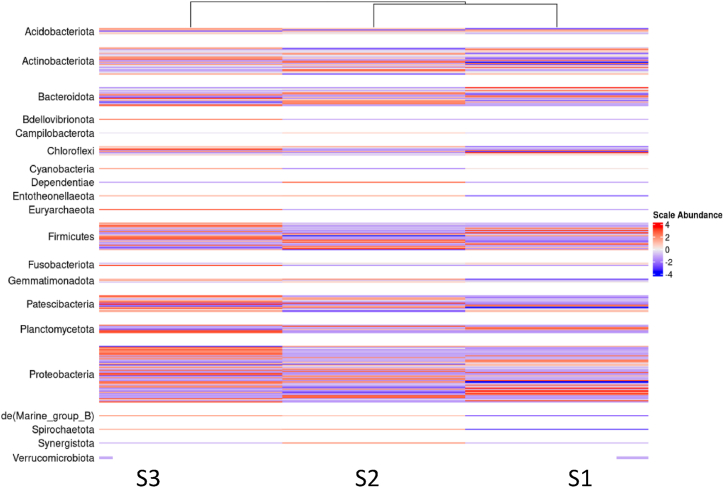
Heatmap of the relative abundance of bacterial genera found in soils never irrigated with wastewater (S1), soils irrigated for 5 years (S2), and soils irrigated for 25 years (S3).

The heatmap shown in Fig. 4 groups the samples according to genus abundance, with colors representing standardized abundances; red indicates a high abundance of the given genus, while blue indicates a low abundance. Genera abundance patterns in the three soils were different and sometimes reversed. The most abundant genera in the soil control (S1) were found to be the least abundant in S3, and *vice versa*. For example, the proportion of sequences belonging to *Aquicella,* a non-cultured *Acidobacteriota, was* lower in soil S1 and increased in soil S2 and S3, passing from 0 %, 3.76 %, and 5.87 % of the total sequences of S1, S2, and S3, respectively. Similarly, the uncultured *Vicinamibacteriaceae* sequences increased from 18.58 %, 22.81 %, and 25.61 % of the total sequences in S1, S2, and S3, respectively. In contrast, the proportion of sequences belonging to the *Lawsonella* genus within *Corynebacteriacea* was more important in S1 soil and decreased in S2 and S3 soil, passing from 2.09 %, 1.34 %, and 0.76 % of the total sequences of S1, S2, and S3, respectively. Similarly, *Sphingomonas* genus sequences within *Sphingomonadacea* accounted for 2.67 %, 2.18 %, and 1.33 % of the total sequences in S1, S2, and S3, respectively.

**Fig. 5:**
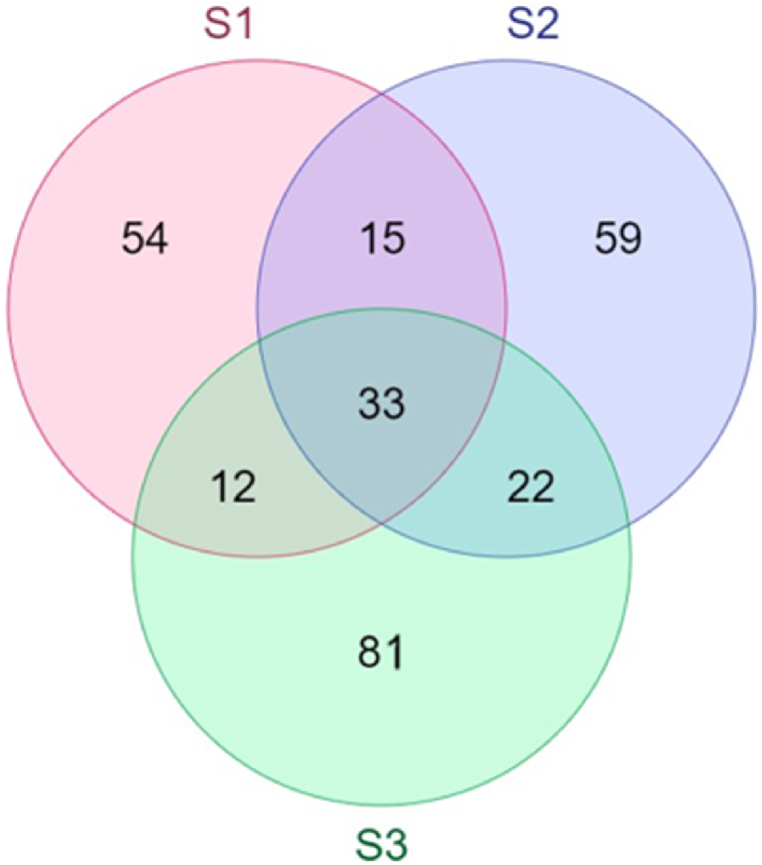
Venn diagram showing the overlap of bacterial communities found in soils never irrigated with wastewater (S1), soils irrigated for 5 years (S2), and soils irrigated for 25 years (S3) based on OTUs.

The Venn diagram (Fig. 5) illustrates the overlap of OTUs found in the three analyzed soil samples. S3 had the highest proportion of unique OTUs (55 %), followed by S2 (45.7 %) and S1 (47.4 %). The most represented ones were OTUs belonging to *Halomonadacea* (3.2 % of all S1 sequences), *Vicinamibacteriaceae* (2.4 % of all S2 sequences), and *Saccharimodacea* (1.7 % of all S3 sequences) for S1, S2, and S3 respectively while12 % of all OTUs were common to the three soils. Within them, we can cite an non-cultivable phylum of *Vicinamibacteriacea* (*Acidobacteriota*) representing 22.6 % of all sequences, as the most represented, and in smaller proportions, an OTU affiliated with *Corynebacterium matruchotii*, *Actinobacteriota* (2.9 %), and *Fusobacterium nucleatum* within the *Fusobacteriota* phylum (2.8 %). Furthermore, there were more shared Operational Taxonomic Units (OTU) between S2 and S3 (8 % of all OTUs) than between S1 and S2 (5.4 %) or between S1 and S3 (4.3 %). The most represented OTUs common to the two soils were an OTU affiliated with *Lentimicrobiacea* (1.2 % of S1 and S2 sequences), an OTU affiliated with *Rotia aera* and *Micrococcacea* (1.1 % of S1 and S3 sequences), and an OTU affiliated with a non-cultivable *Saccharimonadacea* (2.9 % of S2 and S3 sequences).

## 4 Discussion

The reuse of wastewater for irrigation is crucial in agriculture because it is a beneficial solution to water scarcity, especially in arid and semi-arid regions, such as Tunisia. However, these waters can contain many toxic pollutants that interfere with the soil microbiome. In this work, we studied the impact of TWW irrigation duration on soil characteristics, dynamics of the bacterial resistome against antibiotics and heavy metals, and diversity of soil microbial communities irrigated by these waters.

### 4.1 TWW affects soil physicochemical characteristics

The pH measurements of the different soil samples from the Cebela Borj Touil perimeter indicated a basic pH that decreased and became neutral to slightly acidic with the duration of irrigation. This decrease can be explained by the oxidation of organic matter and nitrification of ammonium in the soil. Following the regulations in force, the pH of wastewater should be between 6.5 and 8.5. This trend is counterbalanced by the leaching of active limestone by irrigation water, which is often responsible for soil alkalinity [31]. The results of the soil electrical conductivity monitoring study showed an increase in soils with the longest TWW irrigation duration. This clear elevation is due to the mineral input from TWW being greater than that contained in freshwater (i.e., from the SONEDE, the National Water Distribution Utility treatment). Thus, soils irrigated with TWW showed higher salinity than the control soil (S1). This increase in all irrigated plots was mainly due to the quality of the TWW loaded with salts, the intense evapotranspiration during the summer season, the absorption of water by plants, and the large amount of water applied [32].

### 4.2 TWW affects the bacterial soil community abundance

The results of the soil microflora study of plots irrigated with TWW showed that long-term irrigation induced a significant decrease in soil microbial abundance. This decrease in the number of microorganisms might be due to toxic pollutants still present in TWW, as wastewater treatment in plants is often ineffective for many of them [10,12]. It should be noted that in Tunisia, only primary and secondary treatments have been adopted so far for domestic wastewater treatment.

Thus, after several years of irrigation with TWW, it can be observed that the soils have been impacted, and a large part of the soil microbial community has been affected, leading to a decrease in its abundance. Sensitive microorganisms may be totally decimated, whereas tolerant microorganisms may not affected; their number may even increase because competition with sensitive microorganisms has disappeared or is resilient and growing slowly. In terms of abundance, TWW is probably more harmful to microorganisms than the nutritional benefits it can provide, which is inherent to the overall decrease in microbial biomass [33].

To better understand the resistance potential of telluric microorganisms following TWW irrigation, we were particularly interested in two types of persistent pollutants in soils and water: metallic trace elements and antibiotics.

### 4.3 The impact of irrigation with TWW on the development of heavy metals’ microbial resistance

The results showed that the number of bacteria resistant to Cu, Pb, and Cd was higher with increasing irrigation time using TWW. This result was consistent with the findings of Mertens et al. [34]**.** Thus, there is clear evidence that TWW has an impact on increasing the resistance of soil microbial communities to Cu, Pb, and Cd. However, this increase may correspond to a specific population resistance. For example, it has been shown that community modifications following the presence of Cu lead to an increase in the relative proportions of *Proteobacteria* [35] and *Firmicutes* [36].

Therefore, TWW promotes an increase in heavy metal bacterial resistance by (i) carrying heavy metals, which, through a cumulative effect over time, exerts selection pressure on native soil bacteria, increasing their intrinsic resistance, and (ii) disseminating resistance genes in irrigated soils *via* bacteria carrying mobile genetic elements, such as plasmids [37].

### 4.4 The impact of irrigation with TWW on the development of antibiotics’ microbial resistance

The resistance of microorganisms to antibiotics, specifically TET and AMX, was assessed in this study. The results showed that the number of bacteria resistant to TET (2 mg/L) and AMX (64 mg/L) was higher in soils where the irrigation time with TWW was longer, indicating that bacterial communities from TWW-irrigated soils can tolerate antibiotics more than those from non-wastewater treated soils. AR can be expressed by ARG (with chromosomal location or carried 246 by genetic elements), but it may also result from antibiotrophy, that is, cases where antibiotics represent a carbon source for these bacteria [23]. This is consistent with previous studies indicating that ABs and ARB are carried by TWW [38] and generate other changes, such as physiological and genetic adaptations to antibiotics [39]. Moreover, some authors have shown that very low concentrations of ABs can increase the number of ARB by promoting their adaptive evolution [40], which is consistent with our most plausible explanation regarding what has been occurring when irrigating soils with TWW for several years. This adaptation can also accelerate the degradation of ABs, sometimes to the point of mineralization, thus reducing their persistence in the environment and simultaneously their toxic effects on the environment [38,41,42] by lowering their concentrations.

Bacterial resistance to ABs is the result of co-evolution between organisms that secrete antibiotics and those that adapt to them. This is especially true when it is known that ARG are carried by mobile elements, such as plasmids, integrons, and transposons, which are readily transferred within the soil microbial community [43]. Thus, it is recognized that anthropogenic activities, including the discharge of WWTP effluents, may contribute to the expansion of "reservoirs" of AB resistance [44]. Some authors have shown no changes in microbial activity and very few changes in microbial community composition following irrigation with TWW, supporting the idea that antibiotic concentrations do not appear to exert significant selective pressure on soil microbial communities through environmental protection measures [45]. Our findings also support this hypothesis to a high degree. Specifically, the analysis of bacterial resistance to metal showed that irrigation with wastewater increased their abundance, whereas for AB, this was less obvious. The number of ARB showed significant differences among the three soils studied, suggesting that wastewater irrigation had an impact on the development of antibiotic resistance. As suggested in the review by Slobodiuk et al. [46], the evidence on whether irrigation with treated wastewater increases the prevalence and abundance of antimicrobial resistance (AMR) in soil was mixed according to the literature, highlighting the need to better understand the extent to which AMR is disseminated through this practice. The factors that modify the effect of wastewater irrigation on AMR in soil require further investigation. Key ideas could come from experiments exploring the co-selection of resistance to both antibiotics and heavy metals as well as investigations into antibiotrophy, which could explain the heterogeneity in results between studies.

### 4.5 Co-occurrence of antibiotic- and metal-resistance

The strains isolated in this study showed co-selection between resistance to metals and antibiotics, independently of the nature of the metal or antibiotic. Indeed, bacteria isolated on BHI media containing Pb, Cu, or Cd showed, in 72.3 % of cases, concomitant resistance to at least one antibiotic, TET or AMX. Indeed, the development of resistance to metals often increases bacterial resistance to antibiotics by exerting co-selection pressure [[47], [48]].

The mechanisms underlying metal-induced co-selection for antibiotic resistance are yet to be elucidated, but studies have already shown that they include co-resistance (physical binding of resistance determinants within a single mobile genetic element), cross-resistance (protection of multiple toxins offered by the same resistance determinants), and co-regulation (expression of resistance determinants under the control of the same regulatory pathway) [38].

Furthermore, we observed that multi-tolerance to Cu, Pb, and Cd is significantly associated with multi-AR microorganisms, reinforcing the role of the horizontal transfer of ARG by mobile genetic elements such as integrons [[49], [50]].

### 4.6 Antibiotrophy: treated wastewater irrigation's main consequence

The highest capacity of bacterial microflora to grow in mineral media supplemented with TET and AMX was obtained for the S3 soil community, followed by S2 and S1. This strengthens the fact that some bacteria use ABs as a source of carbon and implies that a long duration of TWW irrigation may confer a selective advantage to bacteria by enhancing their dispersal potential and facilitating ARB introduction and dispersion in the environment. In addition to eliminating sensitive trophic competitors and thus freeing up niches, AB may constitute an exclusive trophic niche for AB-degrading bacteria [51]. Taken together, these results indicate that residual antibiotic concentrations associated with WWTP effluents do not appear to exert sufficiently strong selective pressure to induce the spread of ARG in TWW-irrigated soils and that ARB associated with TWW do not persist in irrigated soils. Even if high numbers of ARB enter soils through TWW irrigation, they are unable to compete or survive in the soil environment, and the high levels of ARB and ARG observed in both non-TWW-irrigated and freshwater-irrigated soils are predominantly associated with resistance by native soil microorganisms [14]. The same authors also agreed that several factors can influence the development of antibiotrophy in wastewater-irrigated soils, including wastewater quality, soil characteristics, and climate. The emergence of antibiotrophy in ARB could encourage their dispersal and survival in the environment and could therefore constitute a health problem. In addition, several challenges and considerations should be considered, including the specificity of the antibiotrophic organisms, efficiency of antibiotic degradation, antibiotic toxicity against microorganisms themselves, and horizontal gene transfer. However, antibiotrophy should benefit the environment by contributing to the self-purification of ecosystems contaminated by antibiotics, reducing antibiotic resistance by reducing the antibiotic load, and cost-effective biological treatment processes.

### 4.7 Bacterial indicators associated with TWW irrigation

16S rRNA metagenomic analysis was carried out to determine the bacterial diversity in soils irrigated for different durations with TWW. A slightly higher abundance was found in S3 soil than in S1 soil.

- *Acidobacteriota* were found to be the predominant phyla in all three soils, but their abundance increased with irrigation duration, possibly linked to soil acidification, which is conducive to their growth [52].
- An increase in *Gammaproteobacteria* was observed from 1 % in S1 soil never irrigated with TWW to 8.2 % in S3 soil after 25 years of TWW irrigation, which is consistent with the results of Broszat et al. [53], indicating that soils irrigated with wastewater over a 100-year period in Mexico showed a 26.7 % increase in the relative abundance of *Proteobacteria.* This was attributed to the high soil carbon availability caused by TWW. *Gammaproteobacteria* are well known to be key players in the global cycle of carbon, nitrogen, and sulfur [54]**.** Commonly detected ARB included fecal bacteria like *E. coli* and *Pseudomonas* [46].
- *Pastescibacteria* was the 4^th^ most frequently detected group in S3, representing 8.8 % of the total sequences. Its number doubled compared with that of soil S1. It is frequently detected in activated sludge and belongs to the “microbial dark matter” as species of this lineage cannot be cultured in laboratories [55]. Uncultured OTUs of filamentous *Saccharimonadia* within this phylum are obligate fermentative bacteria that use heterolactic fermentation pathways and diverse carbon metabolism, including the utilization of oleic acid and amino acids [55].
- *Actinobacteria* were the most prevalent phyla in all three soils, but their numbers decreased with irrigation time. *Actinobacteria* consists of many gram-negative bacteria that play an important role in carbon cycling and the breakdown of environmental chemicals. This reduction is probably explained by its inability to resist various pollutants present in TWW, such as antibiotics.
- *Bacteroidota* and *Firmicutes* were also affected by TWW as their relative abundance decreased (from 11.5 % to 6.7 % and from 7.8 % to 5.2 %, respectively). However, *Firmicutes* has previously been found to be more abundant in TWW-irrigated soils than in non-TWW-irrigated soils [56], as carbon enhances their proliferation. This suggests that wastewater effluents from one wastewater plant differ in qualitative and quantitative composition, thus impacting different microbial growth behaviors.
- The abundance of *Proteobacteria* and *Chloroflexi* remained virtually unchanged between the control and soil irrigated for more than 25 years with wastewater. However, this does not mean that they are not affected by wastewater. Indeed, as these are phyla with high metabolic diversity, some species may be dominated by others that are more competitive within the same group, suggesting stability at the phylum scale.
- Long-term irrigation allows the appearance of new genera, such as *Bdellovibrio*, which is known as a parasitic bacterium of other bacteria, making their hosts resistant to streptomycin [57]. This may shed light on the growing phenomenon of antibiotic resistance. It also allows the disappearance of species belonging to the *Verrucomicrobiota*, which can be explained by the toxicity of pollutants in the treated wastewater.

The unique OTUs retrieved from non-TWW-irrigated soils or, in contrast, those from soils with a long history of irrigation could be considered as bioindicators. Here, we can cite OTUs affiliated with *Halomonadacea* as indicators of no contamination from TWW and those affiliated with *Saccharimonadacea* and *Vicinamibacteracea* as indicators of TWW-originating contamination. Such bioindicators could be useful in the management of soil degradation, and a deeper metagenomic analysis of TWW-irrigated and non-TWW-treated soils is needed to better characterize such tools.

The fact that there is an increasing number of unique OTUs between S1, S2, and S3 and more shared OTUs between S2 and S3 rather than S1 et S2 or S1 and S3 confirms that the observed changes in microbial diversity are the result of TWW soil irrigation.

## 5 Conclusions

This study evidences that irrigation with treated wastewater (TWW) induces significant alterations in soil physicochemical properties, such as soil acidification, increases in salinity and electrical conductivity, shifts in bacterial community composition, and changes in both bacterial abundance and the resistome. The number of ARB tested was significantly different among the soils, with a greater increase in soils irrigated with TWW. Pollutants such as heavy metals (HM) and antibiotics (AB) exert selective pressure on microbial diversity, as evidenced by a decrease in *Actinobacteria*, *Bacteroidetes*, and *Firmicutes,* whereas *Acidobacteria* and *Patescibacteria* increased in TWW-irrigated soils. Additionally, bioindicator species, such as *Halomonadacea, Saccharimonadacea* (indicative of the absence of contamination), and *Vicinamibacteracea* (indicative of its presence), offer valuable insights for assessing soil health.

Our work confirms that although a clear increase in HM resistance of the bacterial community is noted in soils irrigated by TWW, the impact of AB seems to be more complex because (i) antibiotrophy occurs in TWW-irrigated soils, thus mitigating their concentrations, (ii) HM and ATB co-occurrence can exert selection pressure and therefore strengthen resistance, and (iii) mechanisms for AMR naturally exist in native soil communities. In addition, the rate at which an antibiotics act depends on many factors, including its mechanism of action, the presence of resistance mechanisms in bacteria, and environmental conditions. Future work should include high-throughput qPCR of ARG present in TWW, as well as soils that underwent distinct levels of TWW irrigation. Furthermore, Pollution-Induced Community Tolerance (PICT) experiments would help to better understand HM and ARG dissemination in these soils. In addition, Pollution-Induced Community Tolerance experiments would enhance our understanding of HM and ARG dissemination in these soils.

Finally, it should be noted that long-term irrigation (>20 years) unquestionably impacts soil properties and leads to the persistence of pollutants such as HM, whereas for AB-related contamination, it is of greater concern to understand whether ARG are disseminated, and which factors influence this dissemination. The health effects of potential AMR exposure from wastewater-irrigated spoils remain poorly understood. Given this, one can consider soil irrigation with TWW by deciding which impacts are acceptable (or not) in each field and, in the latter case, how to eliminate them at their roots, that is, at the WWTP level.

## CRediT authorship contribution statement

**Amira Yagoubi:** Writing – original draft, Investigation. **Stefanos Giannakis:** Writing – review & editing, Validation, Supervision. **Anissa Chamekh:** Methodology. **Oussama Kharbech:** Formal analysis. **Rakia Chouari:** Writing – review & editing, Supervision, Funding acquisition, Conceptualization.

## Data availability

The data associated with this study have been deposited in the NCBI SRA under the BioProject accession number: PRJNA1048357.

## Ethical approval

Not applicable.

## Funding

This work was supported by the Tunisian Ministry of Higher Education and Scientific Research (LR18ES38). Stefanos Giannakis would like to acknowledge the ARPHILAKE project, “Combating Antibiotic Resistance in Philippine Lakes: One Health upstream interventions to reduce the burden”, which received funding from the Agencia Estatal de Investigación (Spain), Proyectos de Colaboración Internacional (PCI2022-132918), under the umbrella of the “10.13039/100013281JPIAMR - 10.13039/100013281Joint Programming Initiative on Antimicrobial Resistance, and the “DETRAS” Research Project (APOYO-JOVENES-21-UXUKHL-88-WQWWQF), funded by the 10.13039/100012818Comunidad de Madrid through the call “Research Grants for Young Investigators from Universidad Politécnica de Madrid”.

## Declaration of competing interest

The authors declare that they have no known competing financial interests or personal relationships that could have appeared to influence the work reported in this paper.
