# Supplementary material for: Influence of decades-long irrigation with secondary treated wastewater on soil microbial diversity, resistome dynamics, and antibiotrophy development
Source: Heliyon. 2024 Oct 22;10(21):e39666. doi: 10.1016/j.heliyon.2024.e39666 (PMC11544057; doi:10.1016/j.heliyon.2024.e39666)
Supplement: Multimedia component 1 [file mmc1.docx]

**Supplementary material for**

**Influence of decades-long irrigation with secondary treated wastewater on soil microbial diversity, resistome dynamics, and antibiotrophy development**

**Amira YAGOUBI^1,2^, Stefanos GIANNAKIS^2, *^, Anissa CHAMEKH^1^, Oussama KHARBECH^1^, Rakia CHOUARI^1, **^**

*^1^* *University of Carthage, Laboratory of Plant Toxicology and Environmental Microbiology (LR18ES38), Faculty of Sciences of Bizerte, 7021 Bizerte, Tunisia.*

*^2^ Universidad Politécnica de Madrid (UPM), E.T.S. de Ingenieros de Caminos, Canales y Puertos, Departamento de Ingeniería Civil: Hidráulica, Energía y Medio Ambiente, Environment, Coast and Ocean Research Laboratory (ECOREL-UPM), c/ Profesor Aranguren, 3, ES-28040, Madrid, Spain.*

***Corresponding author:** Dr. Stefanos Giannakis, E-mail: [stefanos.giannakis@upm.es](mailto:stefanos.giannakis@upm.es)

****Corresponding author:** Dr. Rakia Chouari, E-mail address: [rakia.chouari@fsb.ucar.tn](mailto:rakia.chouari@fsb.ucar.tn)

**Table S1:** Physicochemical characteristics of the treated wastewater (TWW) effluents used in this study

|  |  |  |
| --- | --- | --- |
| T°C |  | 24.5 |
| pH |  | 8.13 |
| Electrical Conductivity (ms) |  | 3.34 |
| Dissolved Oxygen (mg/L) |  | 4.3 |
| COD (mg/L) |  | 14 |
| Ammonium (NH_4_-N, mg/L) |  | <0.015 |
| Ortho-phosphate (PO_4_, mg/L) |  | 3.53 |
| Nitrite (NO_2_-N, mg/L) |  | 0.028 |
| Nitrate (NO_3_-N, mg/L) |  | 8.54 |
